# Data for constructing insect genome content matrices for phylogenetic analysis and functional annotation

**DOI:** 10.1016/j.dib.2015.12.015

**Published:** 2015-12-17

**Authors:** Jeffrey Rosenfeld, Jonathan Foox, Rob DeSalle

**Affiliations:** a Sackler Institute for Comparative Genomics, American Museum of Natural History, New York, NY, USA; b Division of Invertebrate Zoology, American Museum of Natural History, New York, NY, USA; c Richard Gilder Graduate School, American Museum of Natural History, New York, NY, USA; d Cancer Institute of New Jersey, Rutgers University, New Brunswick, NJ, USA

## Abstract

Twenty one fully sequenced and well annotated insect genomes were used to construct genome content matrices for phylogenetic analysis and functional annotation of insect genomes. To examine the role of e-value cutoff in ortholog determination we used scaled e-value cutoffs and a single linkage clustering approach.. The present communication includes (1) a list of the genomes used to construct the genome content phylogenetic matrices, (2) a nexus file with the data matrices used in phylogenetic analysis, (3) a nexus file with the Newick trees generated by phylogenetic analysis, (4) an excel file listing the Core (CORE) genes and Unique (UNI) genes found in five insect groups, and (5) a figure showing a plot of consistency index (CI) versus percent of unannotated genes that are apomorphies in the data set for gene losses and gains and bar plots of gains and losses for four consistency index (CI) cutoffs.

## Specification Table

**Table:** 

Subject area	*Evolution, phylogenetics*
More specific subject area	*Entomology, Functional Genomics*
Type of data	*Table with html sites for access to insect genomes*
*Phylogenetic matrices in Nexus format*
*Phylogeentic trees in Newick format*
*Lists of FlyBase accessions for functional annotation of genes that are part of CORE genomes and UNIQUE genes*
*Graphs of consistency index (CI) versus number unnannotated genes*
How data was acquired	*Raw data acquired from html download*
*Phylogenetic matrices obtained by single linkage clustering approach*
*Functional Annotation acquired from websites listed below*
*Graphs obtained from phylogenetic analysis*
Data format	*Nexus files; excel spreadsheets; Newick formatted tree files*
Experimental factors	*Not applicable*
Experimental features	*Twenty-one whole insect genomes were filtered using a single linkage clustering approach to generate presence absence matrices for phylogenetic analysis. Lists of gene gains and losses were obtained for specified nodes in the phylogenetic tree using phylogenetic reconstruction approaches. These gene lists were then characterized for functional significance using the websites listed below.*
Data source location	*See*Supplemental Table 1*as described in the Appendix A section of this paper.*
Data accessibility	*Data within this article*

## Value of the data

These data should allow any researcher to

• obtain raw genome sequences from 21 insect taxa for phylogenetic analysis,
• reconstruct phylogenies from the presence/absence matrices to compare to other methods of phylogenetic reconstruction,
• compare specific phylogenetic hypotheses generated by the presence absence matrices of insect genomes with other methods, and
• compare the FlyBase annotations we determined were part of the CORE genome and unique (UNI) in terminal groups in our phylogenetic analysis with other gene lists that might be of significance to insect evolution.

## 1 Data

The data were obtained from html sites listed in Supplemental Table 1, and manipulated to generate a genome content, gene presence/absence matrix for phylogenetic and functional analysis. Several gene presence/absence (genome content) matrices were generated from this process and these are included in this paper in Supplemental Table 2. The trees generated from phylogenetic analysis of these matrices are in Supplemental Table 3.

## 2 Experimental design and methods

The experimental design followed the methods outlined in Rosenfeld et al. [3] and involved the generation of phylogenetic trees to determine specific genes and gene families that have been gained and lost in insect evolution. Lists of gene gains and losses for five major insect groups – Insecta, Hemiptera, Holometabola, Diptera and Hymenoptera – were generated and the functional significance of these lists was assessed.

The following is a list of the steps involved in the generation of

(1) Assembly of 21 insect genomes into a searchable database.
(2) Ortholog determination of genes from these genomes and construction of phylogenetic matrices consisting of presence/absence data.
(3) Phylogenetic analysis of the genome content data (presence/absence matrices).
(4) Character reconstruction of the gains and losses of different genes and gene families for the five insect groups (Insecta, Hemiptera, Holometabola, Diptera and Hymenoptera).
(5) Functional characterization of the genes that are gained and lost in the five insect groups listed above.

The specific methods used in the five steps listed above utilized Phylogenetic Analysis Using Parsimony (PAUP*; [4]) to generate genome content trees. Three metthods were used to do the phylogenetic analyses – Maximum Parsimony with unweighted characters, Maximum Parsimony with Dollo weighting and Maximum Likelihood (using the binGAMMA model). Presence and absence were reconstructed on the phylogenetic trees with PAUP* [4] using the “apolist” command.

Gene lists for the five insect groups (Insecta, Hemiptera, Holometabola, Diptera and Hymenoptera) were then analyzed for functional significance using the following web tools:

UNIPROT Retrieve/ID Mapping – (http://www.uniprot.org/uploadlists/).
g-profiler [1], [2] – (http://biit.cs.ut.ee/gprofiler/).
CateGOrizer [5], [6] – (http://www.animalgenome.org/bioinfo/tools/catego/).

UNIPROT retrieves functional annotations and GO term lists that can then be analyzed using g-profiler [1], [2] for detection of over-representation of GO terms. Lists of over-represented GO terms were then visualized using CateGOrizer [5], [6].

## Figures and Tables

**Supplemental Figure 1:**
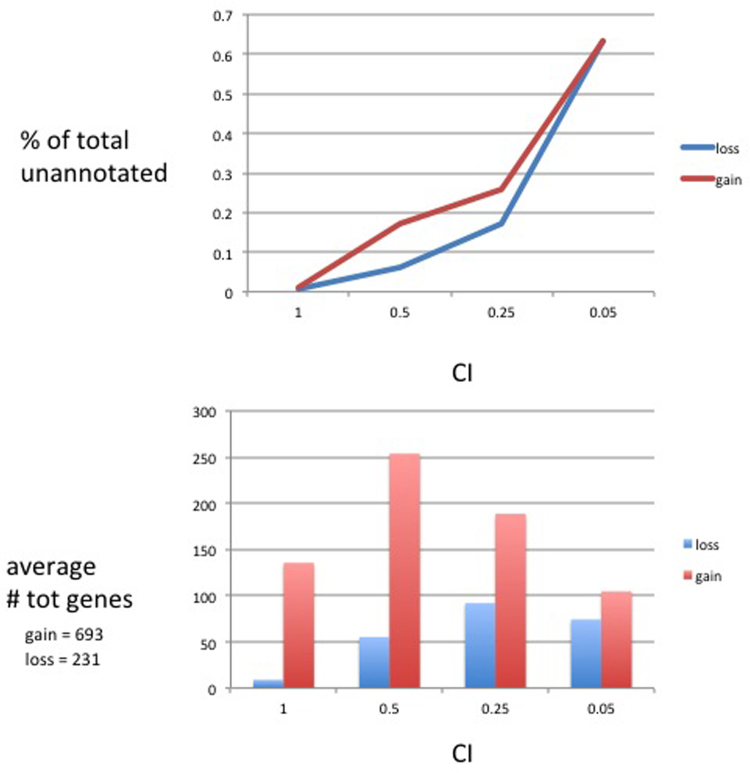
The top figure shows a plot of four consistency index (CI) cutoffs (*X*-axis) versus percent of unannotated genes that are apomorphies in the data set (*Y*-axis) for losses (blue) and gains (red). So for instance, 0% of the genes with consistency index=1.0 in the data set are unannotated. On the other hand, for genes that have consistency index of 0.5, losses show 6% that are unnanaotated and gains show 18% that are unannotated. The bottom figure shows bar plots of gains (red) and losses (blue) for four consistency index (CI) cutoffs.

## Appendix A Supplementary material

Supplemental Table 1: Table showing the accession location of the 21 genomes in this study.

Supplemental Table 2. Nexus file with partitioned phylogenetic matrix for the nine e values examined in this paper for 21 insect taxa.

Supplemental Table 3. File listing the trees generated by ML, uMP and dMP analysis for the seven e values used in this paper.

Supplemental Table 4. List of flybase (FB) accession numbers for CORE and UNI (unique) Apomorphy list genes by taxonomic group.

## Footnotes

Appendix A Supplementary data associated with this article can be found in the online version at doi:10.1016/j.dib.2015.12.015.

## References

[1] J. Reimand, M. Kull, H. Peterson, J. Hansen, J:Vilo: G:Profiler – A Web-based Toolset for Functional Profiling of Gene Lists from Large-scale Experiments, 2007, NAR 35, W193–W200. 10.1093/nar/gkm226 PMC1933153 17478515

[2] Reimand J., Arak T. (2011). J. Vilo: g:Profiler – a web server for functional interpretation of gene lists (2011 update). Nucleic Acids Res..

[3] Rosenfeld J., Foox Jonathon, DeSalle Rob (2015). Insect genome content phylogeny and functional annotation of core insect genomes. Mol. Phylogenet. Evol..

[4] David L. Swofford, {PAUP*. Phylogenetic analysis using parsimony (* and other methods). Version 4.}, 2003.

[5] Zhi-Liang Hu, Jie Bao, James M. Reecy, A Gene Ontology (GO) Terms Classifications Counter, in: Proceedings of the Plant & Animal Genome XV Conference, San Diego, CA, January 13–17, 2007.

[6] Hu Zhi-Liang, Bao Jie, Reecy James M. (2008). CateGOrizer: a web-based program to batch analyze gene ontology classification categories. Online J. Bioinform..

